# Sulforaphane Prevents Testicular Damage in Kunming Mice Exposed to Cadmium via Activation of Nrf2/ARE Signaling Pathways

**DOI:** 10.3390/ijms17101703

**Published:** 2016-10-11

**Authors:** Shu-Hua Yang, Miao Long, Li-Hui Yu, Lin Li, Peng Li, Yi Zhang, Yang Guo, Feng Gao, Ming-Da Liu, Jian-Bin He

**Affiliations:** 1 Key Laboratory of Zoonosis of Liaoning Province, College of Animal Science & Veterinary Medicine, Shenyang Agricultural University, Shenyang 110866, China; yangshuhua0001@126.com (S.-H.Y.); longjlau@126.com (M.L.); yuyang75060@163.com (L.-H.Y.); lilin619619@163.com (L.L.); lipeng79625@163.com (P.L.); sihuo12345@sohu.com (Y.Z.); jessekuo@163.com (Y.G.); gaofengheihei@163.com (F.G.); 2 College of Land and Environmental Sciences, Shenyang Agricultural University, Shenyang 110866, China

**Keywords:** sulforaphane, cadmium, oxidative damage, Nrf2/ARE pathway, testes, mice

## Abstract

Sulforaphane (SFN) is a natural and highly effective antioxidant. Studies suggest that SFN protects cells and tissues against cadmium (Cd) toxicity. This study investigated the protective effect of SFN against oxidative damage in the testes of Kunming mice exposed to cadmium, and explored the possible molecular mechanisms involved. Cadmium greatly reduced the serum testosterone levels in mice, reduced sperm motility, total sperm count, and increased the sperm deformity rate. Cadmium also reduces superoxide dismutase (T-SOD) and glutathione (GSH) levels and increases malondialdehyde (MDA) concentrations. SFN intervention improved sperm quality, serum testosterone, and antioxidant levels. Both mRNA and protein expression of mouse testicular nuclear factor-erythroid 2-related factor 2 (Nrf2) was reduced in cadmium-treated group. Furthermore, the downstream genes of Nrf2, glutathione peroxidase (GSH-Px), γ-glutamyl cysteine synthetase (γ-GCS), heme oxygenase-1 (HO-1), and NAD(P)H:quinone oxidoreductase-1 (NQO1) were also decreased in cadmium-treated group. SFN intervention increases the expression of these genes. Sulforaphane prevents cadmium-induced testicular damage, probably via activation of Nrf2/ARE signaling.

## 1. Introduction

Cadmium chloride (CdCl_2_) is an important industrial raw material. It is widely used in the manufacture of batteries, metal plates, paints, plastics, and alloys. However, cadmium is a known environmental toxin, spread through food intake and smoking. Cadmium poisoning has been reported in individuals engaged in the battery and paint processing industries [1,2]. Testicular injury is a major symptom of CdCl_2_ poisoning. CdCl_2_ destroys blood-testes barrier in rats [3], induces apoptosis of germ cells, and causes testicular edema, hemorrhage, necrosis, and infertility [4,5]. The antioxidant defense system plays a key role in protecting the male reproductive system against oxidative damage.

Natural products have been used to improve reproductive health and prevent diseases triggered by oxidative stress. Natural antioxidants such as onion and garlic extracts [6], *Solanum nigrum* [7], quercetin [8], curcumin [9], grapeseed oil [10], saffron [11], and crocus [12] may ameliorate the reproductive toxicity of cadmium.

Sulforaphane (SFN) is an isothiocyanate derived from the hydrolysis of glucosinolates, present in a large number of cruciferous species such as cabbage, broccoli, and cauliflower [13]. In addition, onions, green onions, eggplants, potatoes, and other vegetables are also rich in sulforaphane [14]. Sulforaphane has antioxidative [15,16,17], antitumor [18], and antimutagenic [19] properties, resulting in bacterial growth inhibition [20] and enhanced immunity [21]. SFN is an endogenous antioxidant, which activates nuclear factor-erythroid 2-related factor 2 (Nrf2) indirectly [22].

The Cap-N-Collar basic leucine zipper (bZip) transcription factor Nrf2 is a master regulator of cellular detoxification responses and redox status [23]. It regulates the expression of more than 200 genes [24], acting as an endogenous antioxidant. Oxidative and electrophilic stimuli trigger Nrf2 activation and transcription of antioxidant enzymes, such as catalase (CAT), NAD(P)H:quinone oxidoreductase (NQO1), heme oxygenase 1 (HO-1), and glutathione *S*-transferase [23]. Nrf2-dependent response leads to cellular detoxification and anti-inflammatory effects. Nrf2-mediated effects play a key role in the antioxidative defense mechanisms of the male reproductive tract [25]. Silencing of Nrf2 leads to reproductive decline following decreased antioxidant capacity and disruption of spermatogenesis with age [26], and studies suggest a positive correlation between Nrf2 transcription and sperm quality [27].

SFN attenuates the oxidative damage in kidneys and heart via regulation of Nrf2 expression [28,29]. Studies suggest that SFN decreases the incidence of apoptosis in mouse testicular cells induced by diabetes types 1 and 2, via activation and upregulation of Nrf2 [30]. Recent studies report epigenetic control by SFN of Nrf2 expression in prostate cancer cells [31]. However, it is unclear whether SFN prevents cadmium-induced testicular injury, via similar mechanisms.

We have created a mouse model of cadmium poisoning, and investigated the protective role of SFN intervention and Nrf2-signaling mechanisms against cadmium toxicity in testicular cells.

## 2. Results

### 2.1. Testis/Body Weight Ratio

Compared with control, testis/body weight ratio of CdCl_2_ group in mice was significantly decreased (*p* < 0.01; Table 1). Mice were treated with SFN, and testis/body weight ratio was not remarkable (*p* > 0.05; Table 1). Compared with CdCl_2_ group, testis/body weight ratio in SFN and CdCl_2_ groups was increased, but not significant (*p* > 0.05; Table 1).

### 2.2. Spermatological Results

**Figure 1:**
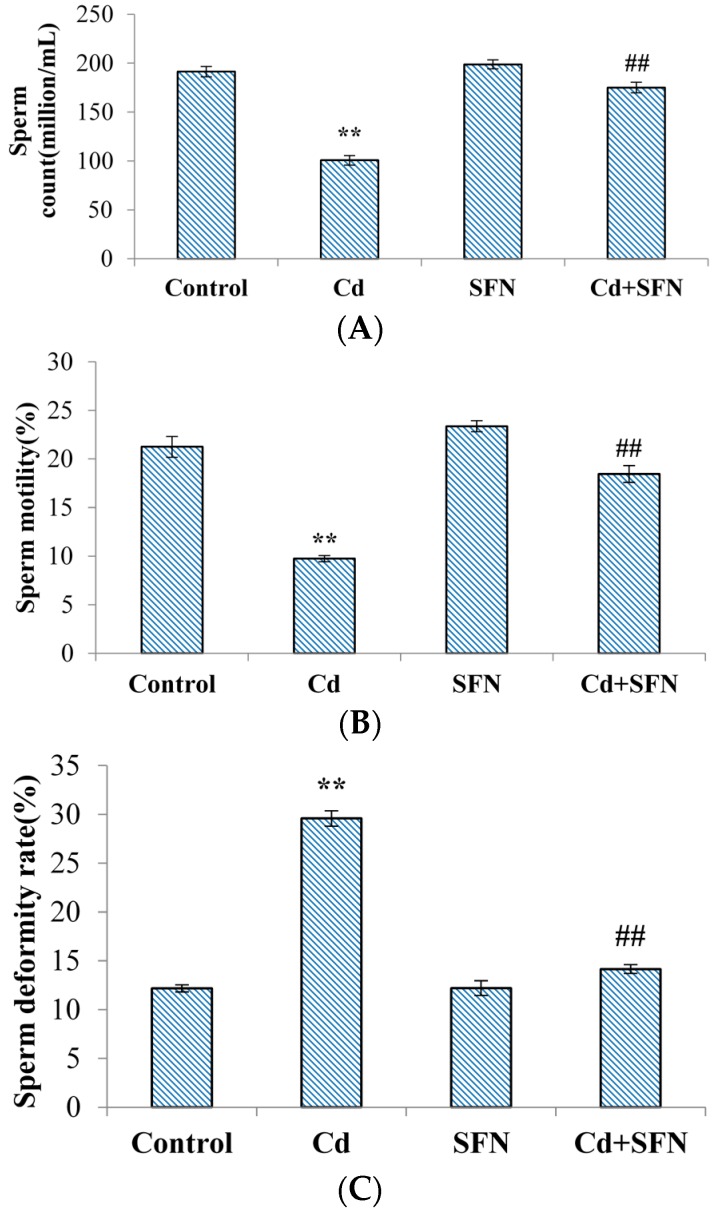
Effect of SFN on sperm quality in mice. (**A**–**C**) After the mice were killed, sperm count (**A**); sperm motility (**B**); sperm deformity (**C**) were examined. Values represent mean ± SEM in each group of 10 mice. * and # denote significant differences; ** *p* < 0.01 vs. control group; ## *p* < 0.01 vs. cadmium group.

Mice treated with CdCl_2_ showed significantly lower sperm motility and count than in the control group (*p* < 0.01; Figure 1A,B). Sperm deformity was significantly higher (*p* < 0.01; Figure 1C). The rates of sperm motility, count and deformity in mice treated with SFN were not significantly different than in the control group (*p* > 0.05; Figure 1A–C). The sperm motility and count in mice treated with SFN and CdCl_2_ were higher than in the animals treated with CdCl_2_ alone (*p* < 0.01; Figure 1A,B), and sperm deformity was significantly decreased (*p* < 0.01; Figure 1C).

### 2.3. Serum Testosterone

**Figure 2:**
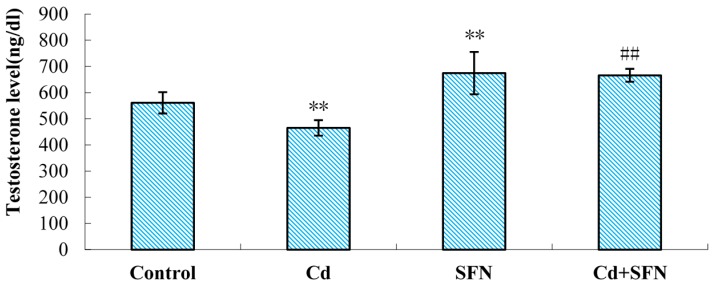
Serum testosterone levels. Values represent mean ± SEM in each group of 10 mice. ** *p* < 0.01 vs. controls; ## *p* < 0.01 vs. cadmium group.

The serum testosterone levels of mice in the CdCl_2_ group were significantly decreased compared with controls (*p* < 0.01; Figure 2). The serum testosterone in mice treated with SFN was significantly higher (*p* < 0.01; Figure 2). Compared with the CdCl_2_ group, the serum testosterone in SFN and CdCl_2_ groups was significantly higher (*p* < 0.01; Figure 2).

### 2.4. Testicular Antioxidant Capacity

The superoxide dismutase (T-SOD) and glutathione (GSH) activities of mouse testicular tissue were significantly lower in the CdCl_2_ group compared with the controls (*p* < 0.01; Table 2). The malondialdehyde (MDA) level was significantly higher (*p* < 0.01; Table 2). The T-SOD and GSH levels in the testicular tissue of SFN mice were significantly increased compared with controls (*p* < 0.05; Table 2). The T-SOD and GSH levels in mouse testicular tissue in the SFN and CdCl_2_ groups were significantly higher than in the CdCl_2_ group alone (*p* < 0.01; Table 2), while the MDA content was significantly lower (*p* < 0.01; Table 2). Significant differences existed between CdCl_2_ group and other groups.

### 2.5. Histopathological Variation

**Figure 3:**
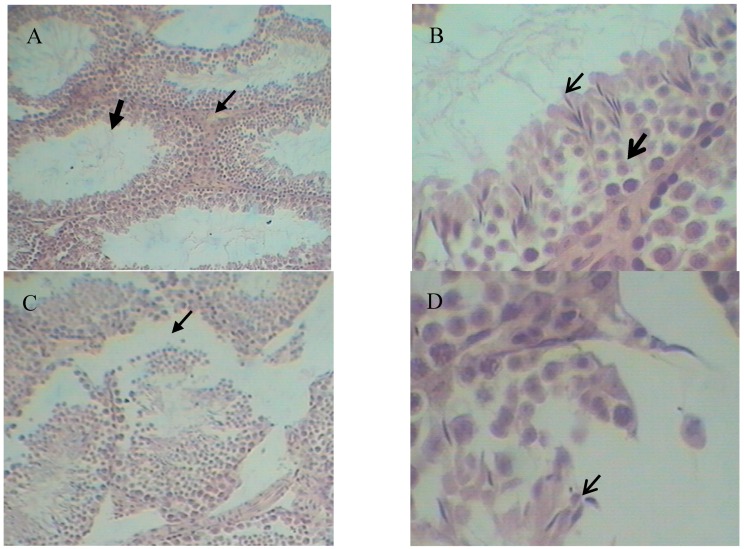

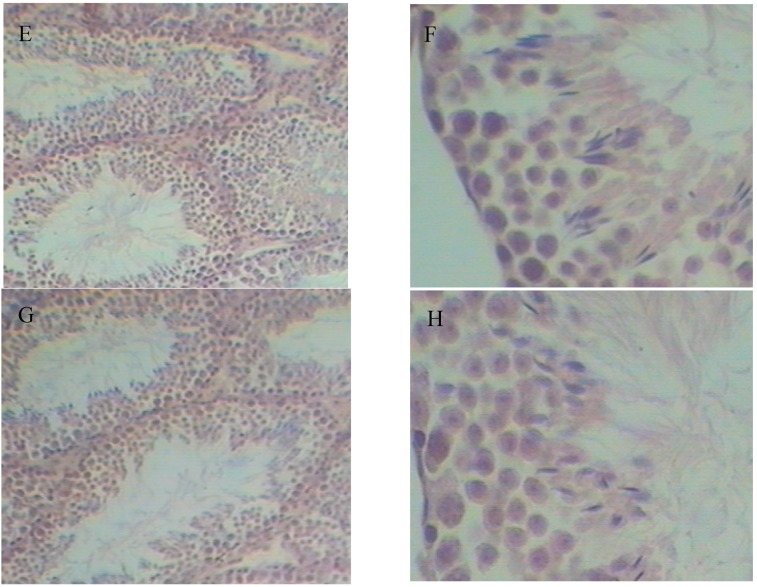
Role of sulforaphane (SFN) in Cd-induced histopathology (original magnification **A**,**C**,**E**,**G** 100×; **B**,**D**,**F**,**H** 400×). (**A**,**B**) Control group; (**C**,**D**) CdCl_2_ group (2.3 mg/kg); (**E**,**F**) SFN group (10 mg/kg); (**G**,**H**) CdCl_2_ (2.3 mg/kg) + SFN (10 mg/kg) group (thin arrow: Leydig cells; thick arrow: seminiferous tubule; open thin arrow: mature sperm; open thick arrow: sperm cell).

Histopathology results are shown in Figure 3. Compared with the control group, the CdCl_2_ group showed damaged seminiferous tubules with a significantly decreased level of sperm cells and mature sperm (Figure 3C,D). The Leydig cells were also fewer (Figure 3C) and the clearance of seminiferous tubule was obviously increased (Figure 3C). No significant differences were seen between the control and SFN groups (Figure 3A,B vs. Figure 3E,F). Compared with the CdCl_2_ group, the SFN and CdCl_2_ combination treatment increased the sperm cell proliferation and maturation as well the number of Leydig cells in the seminiferous tubule (Figure 3G,H). The structure of the seminiferous tubule was more complete, and closely connected with other tubules (Figure 3G).

### 2.6. SFN and Nrf2 Signaling

**Figure 4:**
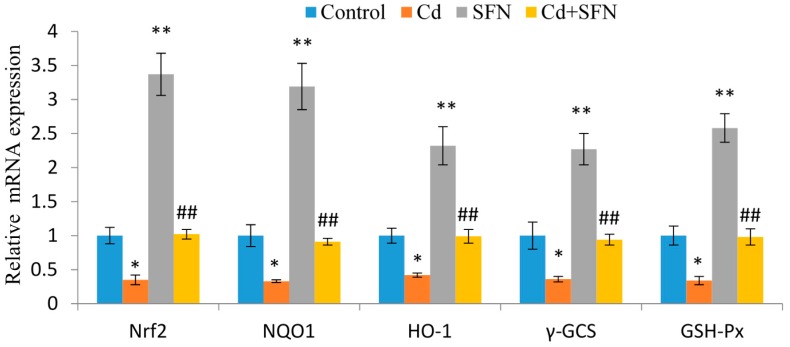
Effects of sulforaphane (SFN) treatment on CdCl_2_-induced transcription of nuclear factor-erythroid 2-related factor 2 (*Nrf2*), glutathione peroxidase (*GSH-Px*), and γ-glutamyl cysteine synthetase (*γ-GCS*) in mouse testis. Values represent mean ± SEM of 10 mice in each group; * *p* < 0.05, ** *p* < 0.01 vs. control group; ## *p* < 0.01 vs. cadmium group.

The role of SFN in the regulation of genes underlying *Nrf2* signaling is shown in Figure 4. The expression of *Nrf2*, *GSH-Px*, *HO-1*, *γ-GCS*, and *NQO1* in the CdCl_2_ group was significantly lower than in the control group (*p* < 0.05). In contrast, SFN substantially enhanced the expression of *Nrf2*, *HO-1*, *γ-GCS*, *GSH-Px*, and NQO1 (*p* < 0.01). Treatment with SFN resulted in a significantly higher increase in the gene expression associated with *Nrf2* signaling than in the CdCl_2_ group (*p* < 0.01), as shown in Figure 4.

### 2.7. Nrf2-Related Protein Expression

**Figure 5:**
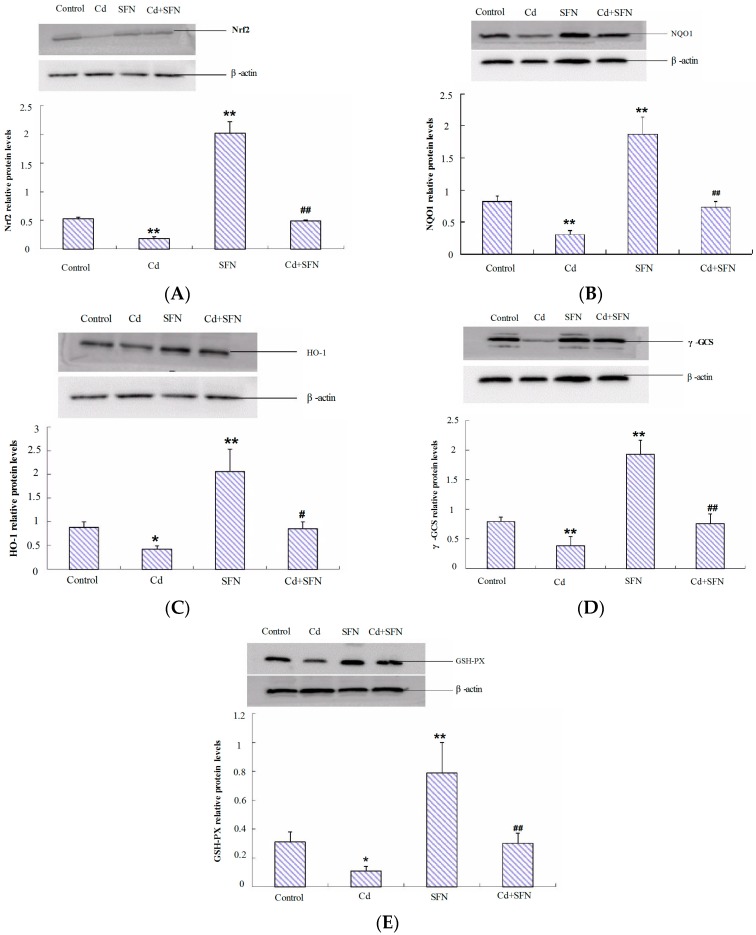
SFN pretreatment and cadmium-induced Nrf2/ARE (antioxidant response element) signaling in mouse testes. (**A**) Nuclear factor-erythroid 2-related factor 2 (Nrf2); (**B**) NAD(P)H:quinone oxidoreductase 1 (NQO1); (**C**) hemeoxygenase-1 (HO-1); (**D**) γ-glutamyl cysteine synthetase (γ-GCS); and (**E**) glutathione peroxidase (GSH-Px). Values represent mean ± SEM in each group of 10 mice; * *p* < 0.05, ** *p* < 0.01 vs. control; # *p* < 0.05, ## *p* < 0.01 vs. cadmium group.

We investigated the role of Nrf2 activation in SFN treatment against cadmium toxicity. We analyzed the expression of Nrf2 and its targets HO-1, NQO1, γ-GCS and GSH-Px in mouse testes. Western blot revealed inhibition by CdCl_2_ on Nrf2 protein expression and also its downstream target proteins NQO1, HO-1, γ-GCS, and GSH-Px. These proteins were significantly downregulated in the CdCl_2_ group, as shown in Figure 5. SFN treatment enhanced the testicular levels of these proteins compared with the control group. SFN pretreatment significantly restored the expression of these proteins (Figure 5), compared with the levels in CdCl_2_ group.

## 3. Discussion

This study investigated the role of sulforaphane in the altered Nrf2 expression and Nrf2-mediated phase 2 enzymatic activity following testicular exposure to cadmium toxicity in mice. Mice were initially treated with cadmium chloride (2.3 mg/kg equivalent to LD_50_ of 10%) and sulforaphane (10 mg/kg). The levels of serum testosterone, total sperm count, and sperm motility were significantly decreased compared with the levels in the control group (*p* < 0.01). The sperm deformity rate was significantly higher in the cadmium-treated group than in the control group (*p* < 0.01). Sulforaphane treatment enhanced the levels of serum testosterone, total sperm count, and sperm motility compared with the cadmium-treated group (*p* < 0.01). Sperm deformity was reduced. hematoxylin and eosin (HE) staining further confirmed the results: abnormal seminiferous tubules, significantly reduced number of mesenchymal and spermatogenic cells, and mature sperm in mice treated with cadmium (Figure 3C,D). Sulforaphane intervention improved the testicular parameters: seminiferous tubules were more closely connected with each other with complete structure, significantly higher number of spermatogenic cells, and more mature sperm and Leydig cells than in the cadmium-treated group (Figure 3G,H). The study revealed that sulforaphane enhanced sperm quality in cadmium-treated mice, and reduced testicular pathology. However, in our study, the mouse testis tissue showed minor histopathological variation, which may be related to the short duration of cadmium exposure. Models investigating germinal cell changes are needed.

Cadmium induces reproductive damage mainly via oxidative stress [32,33,34,35]. Usual parameters such as GSH, SOD, and MDA were used as indices to measure antioxidative stress-induced damage in cells and organs. MDA is often regarded as biomarker of oxidative stress and as a key indicator of tissue damage. It is generated by lipid peroxidation and promotes cellular oxidation [36]. Our results demonstrate higher levels of MDA in the testes of mice exposed to cadmium compared with the control group. Compared with the mice exposed to cadmium, the mice treated with sulforaphane showed lower levels of MDA. SOD is an important antioxidant defense system in cells. The present study found that testicular SOD activity was significantly reduced in the cadmium-treated group, presumably due to oxidative stress, and the findings were consistent with previous studies [37,38]. By contrast, sulforaphane increased the activity of SOD in mouse testis. GSH is a nonprotein thiol compound combined with glutamic acid, cysteine, and glycine, with an active thiol group on the cysteine [39]. Cadmium has strong affinity with thiol; it can form a compound with and then reduce the content of GSH. The reduced γ-GCS protein expression is a key factor contributing to low GSH levels. Sulforaphane treatment significantly increases the level of GSH in mouse testis. Our results show that sulforaphane enhances the antioxidant enzyme activity in mouse testes exposed to cadmium toxicity and attenuates the oxidative damage.

Nrf2 is a key factor in oxidative stress. Nrf2 regulates the expression of antioxidant proteins via interaction with the antioxidant response element (ARE). Under normal conditions, the cytoplasmic Nrf2 mostly combines with Keap1 and is rapidly degraded by the ubiquitin-proteasome [40]. The low concentrations of Nrf2 are inactive. Oxidative stress or stimulation of nucleophilic substances may trigger dissociation of Nrf2 from Keap1 and release free Nrf2. It may also weaken the effect of Keap1-mediated protease on Nrf2 [23]. Nuclear translocation of free Nrf2 and binding with ARE triggers transcription of target genes downstream, including *GSH-Px*, *HO-1*, γ-*GCS*, and *NQO1* [41]. Expression of downstream target genes plays an important role in maintaining redox homeostasis [42]. Our results show that cadmium decreases Nrf2 mRNA levels as well as the transcription of Nrf2-induced downstream target genes (phase 2 genes) (Figure 4), suggesting that cadmium-induced oxidative stress accelerates gene transcription significantly (*p* < 0.01). Previous studies have shown that Cd exposure causes a marked increase in the generation of reactive oxygen species (ROS) that exceed the physiological capacity of the antioxidant enzyme, finally exhausting these enzymes, reducing their activity, and thereby decrease the activity of Nrf2 [43,44]. In our experimental conditions, the endogenous antioxidant system of testis tissue was inhibited at the high doses of cadmium in a short time, resulting in the damage of the testis tissue cells. Treatment with SFN induced Nrf2 at a high expression, activated the Nrf2/ARE signaling pathways, and then induced the expression of the downstream antioxidant mRNA and protein expression.

Nrf2, as a master endogenous antioxidant defenses, plays an essential role in preventing oxidative disruption of the testis [26,27]. Nrf2 protects mouse testis against heat stress [25]. Nrf2 silencing interferes with age-dependent spermatogenesis [26]. Testicular Nrf2 expression in 4-month-old mice with type 1 and type 2 diabetes was significantly reduced [30]. We found that SFN improved the mRNA and protein expression of Nrf2, following cadmium exposure. A previous study demonstrated that autoregulation of Nrf2 via ARE-like elements is present in the proximal region of its promoters [42]. Nuclear accumulation of Nrf2 induces phase 2 genes (GSH-Px, γ-GCS, HO-1, and NQO1) under Cd toxicity, which explains the increased levels of Nrf2 in our study. However, the mechanisms of SFN-induced activation of Nrf2 need to be investigated. Additional studies are needed to investigate the role of SFN in inhibition of Nrf2 ubiquitination and degradation, and to explain the regulatory role of SFN resulting in Nrf2 protein accumulation in cells. However, whether SFN protects reproductive cells against cadmium-induced toxicity via Nrf2/ARE signaling is unknown. Our results suggest that cadmium exposure decreases testicular SOD activity and GSH content. It increases MDA levels, and induces testicular oxidative damage. In addition, this study showed that cadmium alters the expression of genes coding for antioxidant enzymes. Sulforaphane ameliorates reproductive toxicity associated with cadmium exposure, by activating Nrf2 mRNA expression, and regulating the phase 2 antioxidant enzymes and detoxification.

## 4. Experimental Section

### 4.1. Animals

Male Kunming mice aged 9 weeks and weighing 45 ± 4 g were obtained from the Experimental Animal Center of China Medical University, Shenyang, China. Initially, the mice were housed in a room with restricted access, and exposed to 12 h light/dark cycles at a humidity of 40%–60% and a temperature range of 22–24 °C. The animals were provided access to water and diet ad libitum. The mice were exposed to stress and acclimated for 1 week before commencing the study. The experiments were performed in compliance with the European Communities Council Directive dated 24 November 1986 (86/609/EEC), and in accordance with the principles of good laboratory animal care. The experiments were approved by the ethics committee for laboratory animal care at Shenyang Agricultural University, China.

### 4.2. Reagents

SFN was provided by LKT Laboratories (St. Paul, MN, USA). We prepared a stock solution of 100 mg/mL SFN in diethyl sulfoxide and refrigerated at −20 °C. Cadmium chloride (CdCl_2_) with at least 99% purity was purchased from Sinopharm Chemical Reagent Co., Ltd. (Shanghai, China). The testosterone radioimmunoassay kit was supplied by Atomic Transtech Services Limited Co., Ltd. (Beijing, China). The malondialdehyde (MDA), superoxide dismutase (SOD), and glutathione (GSH) levels were measured using reagents provided by Nanjing Jiancheng Bioengineering Institute, China, according to the manufacturer’s protocol. The SYBR green RT-PCR kit was provided by Takara, Japan, and DAPI was purchased from Sigma Aldrich, (St. Louis, MO, USA). The primers for Nrf2, GSH-Px, HO-1, γ-GCS, NQO1, and β-actin were obtained from Sangon Biotech (Shanghai, China). The solution for RNA sample storage and the RNA extraction kits were obtained from Sangon Biotech. The Kits for Revert Aid First Strand cDNA Synthesis were purchased from MBI Fermentas (Burlington, ON, Canada). The mouse anti-Nrf2, anti-γ-GCS, and anti-GSH-Px antibodies were acquired from Santa Cruz Biotechnology (Santa Cruz, CA, USA). The polyclonal anti-HO-1, anti-NQO1 and anti-β-actin antibodies were purchased from Sangon Biotech. We also purchased secondary antibodies conjugated with goat anti-mouse and goat anti-rabbit horseradish peroxidase (HRP) in Beijing Solarbio Science & Technology Co., Ltd. (Beijing, China)

### 4.3. Experimental Design and Treatment

Double-distilled water was administered intraperitoneally to the control group (*n* = 15) daily for 10 days. Double-distilled water combined with a 2.3 mg/kg dose of CdCl_2_ (10% of LD_50_) was administered intraperitoneally to the CdCl_2_ group (*n* = 15) for 10 days. The SFN group (*n* = 15) was intraperitoneally injected with a 10 mg/kg dose of SFN diluted with DMSO and double-distilled water daily, for 10 days. The SFN + CdCl_2_ group (*n* = 15) was similarly injected with a 2.3 mg/kg dose of CdCl_2_ and a 10 mg/kg dose of SFN diluted with DMSO and double-distilled water daily, for 10 days. After the mice were injected with SFN solution for 4 hours, they were injected with CdCl_2_ solution.

Forty-eight hours after stopping the injection of SFN and CdCl_2_, the mice were killed with anesthesia. Subsequently, we extracted blood samples and separated the serum. Finally, the testes isolated from each mouse were stored at −80 °C for further use experimentally.

### 4.4. Sperm Parameters

Analysis of sperm parameters was conducted following Ciftci’s criteria (2012(a,b)) [45,46]. The sperm motility (%), sperm count (million/mL), and the rate of sperm deformity (%) were investigated in this study. The testis and epididymis of mice were collected after mice were killed. Epididymis were placed in the centrifuge tube with 37 °C preheated physiological saline and cut up, put into 37 °C water-bath, and incubated for 20 min; sperm motility was detected visually at a light microscope at 37 °C (400×). Semen were collected, pretreated at 60 °C for 5–10 min, added as a suspension to the hemocytometer, and sperm count observed with the help of a light microscope (200×). Because sperm deformity reflected the sperm quality, analysis of the percentage of abnormal sperm was necessary. The slides were stained with 2% eosin. Suspension (500 µL) was put into the centrifuge tube, added to 50 µL 2% eosin. After 1–2 min, smear slides were made, properly heated on the alcohol lamp and dried. Slides were fixed with formaldehyde after drying, then observed under a light microscope (400×). A total of 300 sperm cells per slide were examined.

### 4.5. Determination of Serum Testosterone

Serum testosterone was measured using a radioimmunoassay (RIA) kit (125I RIA kit, Beijing, China), according to the manufacturer’s protocol. Radioactivity was determined using an automated gamma counter. All samples were analyzed in duplicate in a single assay to avoid interassay variation.

### 4.6. Antioxidant Parameters

We analyzed the oxidant levels in the mouse testes based on MDA content, using a standard assay. We measured the antioxidant enzyme levels in the testes by analyzing the activities of T-SOD and GSH content, using standard commercial kits, according to the manufacturer’s instructions.

### 4.7. Histopathology

Testicular samples were initially fixed in 10% formalin followed by staining with hematoxylin and eosin, and visualization under a photomicroscope.

### 4.8. Gene Expression

Total RNA was extracted from the testicular tissues using TRIzol. The cDNA was synthesized from 1 µg of total RNA using PCR (Sangon Biotech). The primers (Nrf2: NM_010902.3; GSH-Px: X03920.1; HO-1: NM_010442.2; γ-GCS: U85414.1; NQO1: NM_008706.5; and β-actin: BC138614.1) were obtained from Sangon Biotech. The fold differences between samples were determined using the comparative *C*_t_ method and normalized to an internal standard (β-actin). Real-time PCR was performed as described previously [47].

### 4.9. Western Blot

The testes were lysed with RIPA lysis buffer containing 1 mM PMSF. Protein was estimated using the bicinchoninic acid (BCA) method. Total protein (50 µg) was fractionated on 10% SDS-PAGE gels, transferred electrophoretically onto a polyvinylidene difluoride membrane (Bio-Rad, Hercules, CA, USA), and blocked with 5% nonfat dry milk in TBST buffer for 2 h. Membranes were incubated overnight at 4 °C with anti-Nrf2 (1:500, Santa), anti-GSH-PX (1:500, Santa), anti-γ-GCS (1:500, Santa), anti-HO-1 (1:300, Sangon Biotech), and anti-NQO1 (1:1000, Sangon Biotech) antibodies. Membranes were incubated with HRP-conjugated secondary antibodies, and chemiluminescence (Super ECL Plus, Applygen, Beijing, China) was analyzed using DNR Bio Imaging Systems and analysis software. Membranes were re-incubated with anti-β-actin (1:2000, Sangon Biotech), for normalization, followed by densitometry [44,47].

### 4.10. Statistical Analysis

Data were expressed as mean ± standard error (X ± SE). One-way ANOVA was used to assess the significance of differences between the mean values. Multiple pair-wise comparisons were conducted using a Student-Newman–Keuls (SNK) post hoc test or the least significant difference (LSD). SPSS 13.0 was used for all statistical analyses.

## 5. Conclusions

In summary, SFN reduces the oxidative damage triggered by cadmium exposure via Nrf2/ARE signaling. In conclusion, this is the first study investigating the role of SFN in combating cadmium-induced reproductive toxicity via activation of Nrf2/ARE signaling pathways in mice.

## Figures and Tables

**Table 1:** Effects of sulforaphane (SFN) on mice body and testis weights and testis/body weight ratio following cadmium exposure.

Group	Body Weight (g)	Testis Weight (mg)	Testis/Body Weight Ratio (mg/g)
Control	45.57 ± 1.69	304.47 ± 10.04	6.68 ± 0.10
CdCl_2_ (2.3 mg/kg)	42.75 ± 1.42	193.65 ± 20.30 **	4.60 ± 0.64 **
SFN (10 mg/kg)	40.43 ± 0.89 *	278.27 ± 11.84	6.88 ± 0.33
CdCl_2_ (2.3 mg/kg) + SFN (10 mg/kg)	40.35 ± 1.89	223.12 ± 28.75	5.48 ± 0.53

Values represent mean ± SEM in each group of 10 mice; * denote significant differences; * *p* < 0.05; ** *p* < 0.01 vs. control group.

**Table 2:** Effects of sulforaphane on antioxidant levels following cadmium exposure.

Group	T-SOD (U/mgprot)	GSH (mg/gprot)	MDA (nmol/mgprot)
Control	86.67 ± 3.43	142.47 ± 2.93	12.69 ± 1.07
CdCl_2_ (2.3 mg/kg)	43.23 ± 12.76 **	33.63 ± 10.15 **	65.83 ± 1.07 **
SFN (10 mg/kg)	119.92 ± 6.41 *	162.14 ± 7.68 *	11.21 ± 0.69 *
CdCl_2_ (2.3 mg/kg)+ SFN (10 mg/kg)	104.61 ± 6.10 ^##^	118.09 ± 10.15 ^##^	19.17 ± 4.17 ^##^

MDA: malondialdehyde; T-SOD: superoxide dismutase. GSH: glutathione. Values represent mean ± SEM in each group of 10 mice. *^,#^ denote significant differences; * *p* < 0.05, ** *p* < 0.01 vs. control group; ^##^ *p* < 0.01 vs. cadmium group.
